# Supplementary material for: A Fresh Perspective on Examining Population Emotional Well-Being Trends by Internet Search Engine: An Emerging Composite Anxiety and Depression Index
Source: Int J Environ Res Public Health. 2024 Feb 9;21(2):202. doi: 10.3390/ijerph21020202 (PMC10888063; doi:10.3390/ijerph21020202)
Supplement: Supplementary file 1 [file ijerph-21-00202-s001.zip › ijerph-2831893-supplementary.pdf]

Supplemental Material

Supply: Table 1 The Summary of Textual Materials

Supply: Table 2 The Public Opinion Dictionary and the Word Importance

Supply: Table 3 Descriptive Statistics for Explanatory Variables

Supply: Table 4 The Meaning of the Explanatory Variables

Supply: Table 5 Stratification of Regions by Per Capita GDP

Supply: Method 1 Word Correlation and Importance

Supply: Table S1 The Summary of Textual Materials

Type	Platform (Detailed Sources)	Amount
Anxiety	Douban ("Anxiety Disorder" Group)	**1,502**
	Weibo ("Anxiety" Super Talk Post)	**151**
	Zhihu ("Anxiety" Q&A Post)	**702**
Depression	Douban ("Treatment of Depression" Group)	**3,882**
	Weibo ("Depression" Super Talk Post)	**823**
	Zhihu ("Depression" Q&A Post)	**699**

Supply: Table S2 The Public Opinion Dictionary and the Word Importance

Anxiety Dictionary	Importance	Depression Dictionary	Importance
Anxiety	1.000	*Depression*	1.000
Generalized anxiety disorder	0.809	*Mental illness*	0.864
Generalized anxiety difficulty	0.806	*Mental disorder*	0.855
Neurasthenia	0.793	*Bipolar disorder*	0.837
Severe insomnia	0.780	*Schizophrenia*	0.831
Psychological anxiety	0.697	*Symptoms of depression*	0.802
Mood disorder	0.668	*Individuals with depression*	0.795
Extreme anxiety	0.637	*Insomnia*	0.773
Mental tension	0.615	*Psychologist*	0.721
Separation anxiety	0.610	*Autistic*	0.654
Irritable	0.600	*Mental hospital*	0.633
Irritability	0.588	*Mental health*	0.585
Anxious	0.551	*Melancholy*	0.534
Insecurity	0.536	*Can't sleep*	0.527
High pressure	0.533	*Self-abasement*	0.518
Worrying and nervous	0.520	*Emotional*	0.485
Negative emotion	0.498	*Sad*	0.440
Upset	0.485	*Meaning of living*	0.425
Terribly upset	0.470	*Uncomfortable*	0.419
Negative	0.464	*Cry*	0.410
Depressed	0.452	*Concern*	0.391

Note: For the convenience of reading, we only show the results of Chinese-English translation.

Supply: Table S3 Descriptive Statistics for Explanatory Variables

Explanatory Variables	Code		Mean	Std	Min	Max
Economic Development						
Disposable Income	DI	overall	2.926	1.224	1.363	7.802
		between	-	1.156	1.828	6.619
		within	-	0.399	1.737	4.109
Consumer Price Index	CPI	overall	107.515	5.589	89.238	123.901
		between	-	1.935	104.582	114.383
		within	-	5.362	87.342	122.005
Consumer Goods Retail Index	CGR	overall	1.071	0.067	0.792	1.265
		between	-	0.029	0.994	1.116
		within	-	0.060	0.789	1.233
Import-Export Index	IE	overall	1.088	0.217	0.429	2.005
		between	-	0.155	0.857	1.862
		within	-	0.197	0.494	2.058
Medical Burden						
Average Cost of Patients	ACP	overall	5.400	1.912	2.980	13.920
		between	-	1.843	3.260	12.14
		within	-	0.500	3.980	7.320
Proportion of Health Expenditure	RHE	overall	7.872	1.926	3.000	12.800
		between	-	1.813	3.340	11.400
		within	-	0.713	5.912	10.212
Risk Degree of Medical Expenditure	RD	overall	0.114	0.025	0.054	0.174
		between	-	0.023	0.060	0.158
		within	-	0.009	0.090	0.139
Rate of Unemployment	RU	overall	3.140	0.619	1.300	4.610
		between	-	0.543	1.888	4.130
		within	-	0.340	2.172	4.482

Note. "overall" represents the statistics of all regions, "within" is the intragroup statistics of each region, and "between" is the intergroup statistics of each region.

Supply: Table S4 The Meaning of the Explanatory Variables

**Explanatory Variables**	**Meaning**
Disposable Income	Refers to the sum of a household's final consumption expenditure and savings, representing the income that can be used for discretionary purposes in units of 10,000 Chinese yuan.
Consumer Price Index	A relative measure of the price change trend and extent of consumer goods and services purchased by both urban and rural residents within a specified period of time.
Consumer Goods Retail Index	Refers to the number of physical goods sold directly by enterprises to individuals and social groups for non-production and non-business purposes through transactions, including the income generated from providing catering services.
Import-Export Index	Represents the ratio of the total value of all actual import and export commodities in a given region during a certain period, expressed in currency, relative to the previous period.
Average Cost of Patients	Refers to the average cost of medical treatment incurred by both inpatients and outpatients, measured in units of 1,000 Chinese yuan.
Proportion of Health Expenditure	Represents the ratio of a family's health care spending to their total household expenditure.
Risk Degree of Medical Expenditure	Refers to the level of risk a family may face when encountering financial difficulties due to medical expenses.
Rate of Unemployment	Represents the proportion of the labor force that remains jobless among the employed population meeting all employment conditions during a specific period.

Supply: Table S5 Stratification of Regions by Per Capita GDP

L-PCG	H-PCG
Gansu; Guangxi; Guizhou; Hebei; Jilin; Heilongjiang; Qinghai; Shanxi; Xizang; Xinjiang; Yunnan; Jiangxi; Henan; Sichuan; Hainan; Anhui; Ningxia; Hunan; Liaoning	Hubei; Chongqing; Neimeng; Shandong; Guangdong; Fujian; Zhejiang; Tianjin; Jiangsu; Shanghai; Beijing; Shaanxi

Supply: Method S1 Word Correlation and Importance

We used the Continuous Bag-of-Words (CBOW) model to measure the strength of association between each word in the POD and the target words. The CBOW model is a feed-forward neural network language model (Mikolov et al., 2013) and has gained widespread usage in many natural language processing applications, including sentiment analysis, semantic similarity calculation, and natural language reasoning. For example, the word vectors computed by CBOW model could be used as features in a classifier for detecting sarcasm in online comments or incorporated into text classification tasks (Liu et al., 2018; Basiri et al., 2021). The following figure is the architecture of the CBOW model. After applying the CBOW model on the collected texts, we obtained the probability for each word in POD. A higher probability indicated a stronger association between the word and its target word.

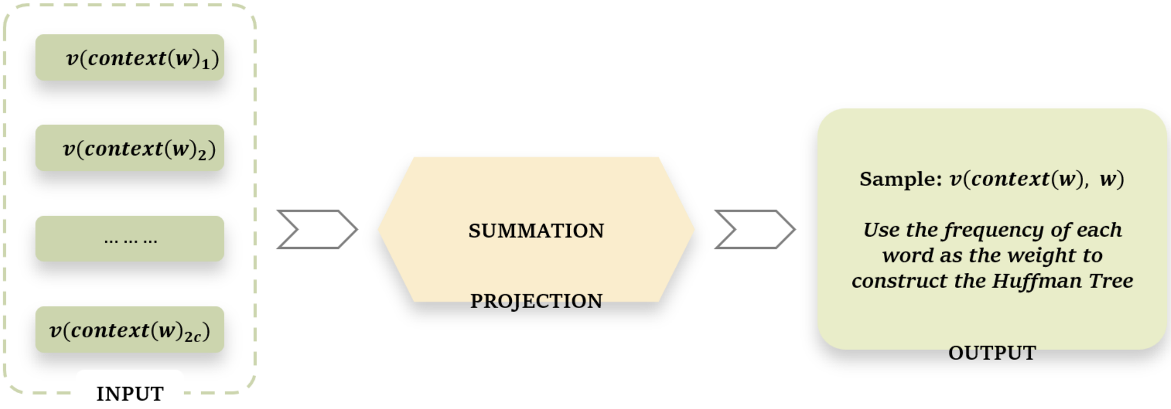

The input layer of CBOW model contains the word vector $v(Context(w)_i) \in R^m$ of 2c words in $Context(w)$, and the m represents the length of the word vector. In the projection layer, the vectors of the input layer are summed and accumulated. The output layer uses the words that appear in the corpus as leaf nodes to generate a binary tree, and uses the frequency of each word as the weight to construct the Huffman tree.
